# Self-Efficacy Effects on Symptom Experiences in Daily Life and Early Treatment Success in Anxiety Patients

**DOI:** 10.1177/21677026231205262

**Published:** 2024-05-14

**Authors:** Christina Paersch, Dominique Recher, Ava Schulz, Mirka Henninger, Barbara Schlup, Florian Künzler, Stephanie Homan, Tobias Kowatsch, Aaron J. Fisher, Andrea B. Horn, Birgit Kleim

**Affiliations:** 1 Experimental Psychopathology and Psychotherapy, Department of Psychology, University of Zurich; 2 Department of Psychiatry, Psychotherapy, and Psychosomatics, University Hospital of Psychiatry, University of Zurich; 3 University Hospital of Child and Adolescent Psychiatry and Psychotherapy, University of Bern; 4 Psychological Methods, Evaluation, and Statistics, Department of Psychology, University of Zurich; 5 Institute for Implementation Science in Health Care, University of Zurich, Zurich, Switzerland; 6 School of Medicine, University of St. Gallen, St.Gallen, Switzerland; 7 Centre for Digital Health Interventions, Department of Management, Technology, and Economics at ETH Zurich, Zurich, Switzerland; 8 Department of Psychology, University of California, Berkeley; 9 University Research Priority Program “Dynamics of Healthy Aging,” Department of Psychology, University of Zurich

**Keywords:** self-efficacy, anxiety, hope, psychophysiological arousal, transdiagnostic, cognitive behavior therapy, CBT, treatment change, ecological momentary assessment

## Abstract

Self-efficacy is a key construct in behavioral science affecting mental health and psychopathology. Here, we expand on previously demonstrated between-persons self-efficacy effects. We prompted 66 patients five times daily for 14 days before starting cognitive behavioral therapy (CBT) to provide avoidance, hope, and perceived psychophysiological-arousal ratings. Multilevel logistic regression analyses confirmed self-efficacy’s significant effects on avoidance in daily life (odds ratio [*OR*] = 0.53, 95% confidence interval [CI] = [0.34, 0.84], *p* = .008) and interaction effects with anxiety in predicting perceived psychophysiological arousal (*OR* = 0.79, 95% CI = [0.62, 1.00], *p* = .046) and hope (*OR* = 1.21, 95% CI = [1.03, 1.42], *p* = .02). More self-efficacious patients also reported greater anxiety-symptom reduction early in treatment. Our findings assign a key role to self-efficacy for daily anxiety-symptom experiences and for early CBT success. Self-efficacy interventions delivered in patients’ daily lives could help improve treatment outcome.

Self-efficacy is a key construct in behavioral science with significant impact on mental health, including psychopathology symptoms and treatment outcomes (Bandura, 1988). It refers to individuals’ belief in their ability to achieve goals and exercise control over their environment, thoughts, emotions, and actions (Bandura, 1994). Self-efficacy has been investigated with a focus on between-persons effects and group differences and its impact on psychopathology-symptom severity and remission (Jakubovski & Bloch, 2016; Thomasson & Psouni, 2010). Here, we expand on these between-groups differences and investigate self-efficacy’s associations with intraindividual change in avoidance, hope, and perceived psychophysiological arousal in the daily life of patients with anxiety disorders and early treatment response.

Research on various psychological disorders has suggested that psychological, behavioral, and emotional outcomes change on a daily and sometimes even on an hourly basis in anticipatory anxiety or panic frequency (DeVries, 1992; Helbig-Lang et al., 2012). Such studies have led to a better understanding of therapy-relevant psychological processes. For instance, although anticipatory anxiety significantly increased after panic attacks in a daily diary assessment in panic patients, patients’ perceived ability to cope with panic mitigated these effects (Helbig-Lang et al., 2012). Such findings call into question the results of cross-sectional studies that have not capture fluctuations over time and context (Fisher et al., 2018). In fact, because there is no mathematical or statistical basis for assuming that between-persons effects should correspond with within-persons effects (Molenaar, 2004), Fisher et al. (2018) argued that clinically meaningful between-persons findings should be directly interrogated at the individual level. In the current study, we thus aimed to investigate the within-persons effect of self-efficacy on avoidance, hope, and perceived psychophysiological arousal using ecological-momentary-assessment (EMA) methods and their association with early symptom changes during psychotherapy.

Self-efficacy and the perceived belief in one’s ability to cope with adverse events and potential threats have been related to arousal and psychophysiological reactions. Greater self-efficacy has been associated with a lower cortisol response to stress (Nierop et al., 2008) and reduced autonomic arousal after mental challenges (Sanz et al., 2006). Lower self-efficacy, on the other hand, was associated with increased catecholamine secretion in individuals with phobia (Bandura, 1988; Maddux, 1990). Self-efficacy has also been increased experimentally by personal mastery-experience inductions, and such increases were associated with greater immunological functioning during exposure to feared situations (Bandura et al., 1982; Wiedenfeld et al., 1990).

In psychotherapy, self-efficacy has been associated with anxiety symptom change over the course of cognitive behavior therapy (CBT; Fentz et al., 2013; Jakubovski & Bloch, 2016); self-efficacy increased during CBT, which predicted symptom reduction (Casey et al., 2005). Decreased self-efficacy before treatment, on the other hand, may hamper treatment effects on fear and avoidance (Gallagher et al., 2013). Thus, enhancing adaptive perceptions of personal control during CBT (i.e., self-efficacy) may improve treatment outcomes in anxiety disorders (Gallagher et al., 2014; Meuret et al., 2010). In accord with these findings, a recent meta-analysis identified self-efficacy-related cognitions concerning one’s ability to cope with threatening situations or sensations as vital mechanisms of therapeutic change (Breuninger et al., 2019). However, these conclusions are so far mostly based on between-persons associations between self-efficacy and anxiety symptoms and rely on retrospective self-report that may differ in real life. Panic attacks and anticipatory anxiety varied, for instance, between cross-sectional and repeated real-time assessments within patients, highlighting the need for further within-persons assessment studies (Craske et al., 1995; Helbig-Lang et al., 2012).

Although self-efficacy refers to the specific expectation that one is capable of following through on a plan of action, individuals may also endorse a more general belief that things may change for the better regardless of their own actions. Such hope represents a positive expectancy that has been applied to uncertainty and potential threatening situations (Snyder et al., 1991). Positive expectations significantly affect patient motivation and action, often leading to better treatment participation and treatment outcome (Constantino et al., 2011; Glass et al., 2001; Greenberg et al., 2006). Such downstream benefits have been observed for patient-held beliefs and expectations about subsequent symptom change and for perceived treatment efficacy (Constantino et al., 2011; Meyerhoff & Rohan, 2016). Similar to self-efficacy, hope has been suggested as a mechanism of change across CBT treatment in anxiety disorders (Gallagher et al., 2020). Their joint effects have not yet been investigated, however, and it has been suggested that self-efficacy might initiate hope (Rand, 2017; Scioli et al., 1997), but empirical findings on self-efficacy’s and hope’s potential interaction and cumulated effects on psychopathology and treatment outcome are scarce (Gallagher et al., 2019). A better understanding of such expectations and their effects on daily anxiety symptoms and their impact on change during treatment could be used to harness their effects to improve treatment. CBT is currently the treatment with the strongest evidence for enduring symptom change in anxiety disorders. Unfortunately, a substantial number of patients drop out early from such therapies, remain symptomatic, or are unable to sustain cognitive and behavioral changes. Thus, existing therapies fail to bring about complete recovery and leave significant room for improvement (Holmes et al., 2014). Identifying predictors and underlying moderators and mechanisms of treatment response is warranted to provide modifiable targets for more effective interventions.

## The Current Study

In the present study, we used EMA to collect intensive repeated measures data with enough person-level statistical power to investigate the effects of self-efficacy on anxiety symptoms in patients’ daily lives. We evaluated effects of self-efficacy on avoidance, hope, and perceived psychophysiological arousal and early symptom changes during transdiagnostic CBT treatment. Given extant between-persons findings in the literature, we first examined whether higher levels of anxiety would be associated with greater avoidance, less hope, and greater perceptions of physiological arousal. Substantiating these processes at the within-individuals level would corroborate existing mechanistic assumptions in the psychotherapy literature. We hypothesized specific directional relationships such that higher levels of self-efficacy would be associated with lesser avoidance, greater hope, and lower perceived psychophysiological arousal. We expect cross-level interactions between self-efficacy and anxiety such that individuals higher in both will exhibit lesser avoidance, greater hope, and less perceived psychophysiological arousal during their daily lives. Finally, we hypothesized that patients with higher levels of self-efficacy would show greater reduction in anxiety and depression in the early phase of CBT treatment. We report how we determined our sample size, all data exclusions, all manipulations, and all measures in the study.

## Method

### Transparency and openness

Data and code for this article are available at https://osf.io/54tpc/. The app for EMA assessments was developed for the Apple iOS platform and for Android smartphones with MobileCoach (www.mobile-coach.eu), an open-source software platform (Filler et al., 2015; Kowatsch et al., 2017). Data and code are available on OSF: https://osf.io/54tpc/.

### Participants

Patients were recruited from the public using newspaper articles, a study website, online platforms, mailing lists from public institutions, and flyers. The eligibility criteria were a primary diagnosis of anxiety disorder from the fifth edition of the *Diagnostic and Statistical Manual of Mental Disorders* (Lecrubier et al., 1997; i.e., panic disorder with or without agoraphobia, generalized anxiety disorder, social anxiety disorder, anxiety disorder not otherwise specified, adjustment disorder with anxiety, adjustment disorder with mixed anxiety and depressed mood, specific phobia with severe impairment), age between 18 and 65 years, and fluent German speaking. Patients were excluded if they were currently undergoing concomitant psychotherapy treatment; if they had a current or past diagnosis of a schizophrenia spectrum disorder or bipolar disorder, current suicidal ideation or acute suicidality, current substance or alcohol dependence or abuse, or Cluster A or B personality disorder; or if there were any medical contraindications that impede thorough exposure, including cardiovascular diseases or autoimmune diseases. If on medication, the patients needed to be stable in their dose for at least 3 months and to agree on maintaining dosage and frequency throughout the study. Ethical approval was granted by the cantonal ethics committee of Zurich, and all patients provided written informed consent.

Up until April 2020, 69 patients were included in the clinical trial. For the current study, we excluded one patient because of missing self-efficacy data at baseline and another two because of insufficient compliance in the EMA data (< 10%). The final sample for the analysis of the EMA data comprised 66 patients (72.7% women). The patients were between 18 and 58 years old (*M* = 32.5 years, *SD* = 11.1). Most of the patients (98.4%) were Swiss or European. With a median income of 60,000 Swiss francs, the sample is broadly representative of the population of Switzerland (Jaquet et al., 2019) considering that 27% of the sample were still in training. Twenty-nine patients (43.9%) were diagnosed with social phobia, 21 patients (31.8%) were diagnosed with panic disorder with agoraphobia, one patient (1.5%) was diagnosed with panic disorder without agoraphobia, 12 patients (18.2%) were diagnosed with generalized anxiety disorder, and three patients (4.5%) were diagnosed with specific phobia. Thirty-seven patients (59.1%) were diagnosed with at least one comorbid disorder. Four patients (6.0%) reported taking psychotropic medication, such as a selective-serotonin-reuptake-inhibitor (*n* = 3, 4.5%) or a noradrenalin-dopamine-reuptake-inhibitor (*n* = 1, 1.5%). To analyze the impact of self-efficacy on early changes during therapy, 50 patients (73.5%) were randomly assigned to the therapy group (UP), one patient dropped out of therapy after the first session, and one patient had a delayed start of therapy because of the outbreak of the COVID-19 pandemic. Hence, a subsample comprising 48 patients was used for analysis on early treatment change.

A power analysis for multilevel data using a longitudinal study design (Diggle et al., 2002) suggested that a minimum of 45 subjects was needed to detect the true effect with an 80% probability, an intraclass correlation coefficient of .55 based on previous work on social anxiety (Kashdan et al., 2014), and 70 assessments (five assessments on 14 days) at an alpha level of .05.

### Procedure

**Fig. 1.**
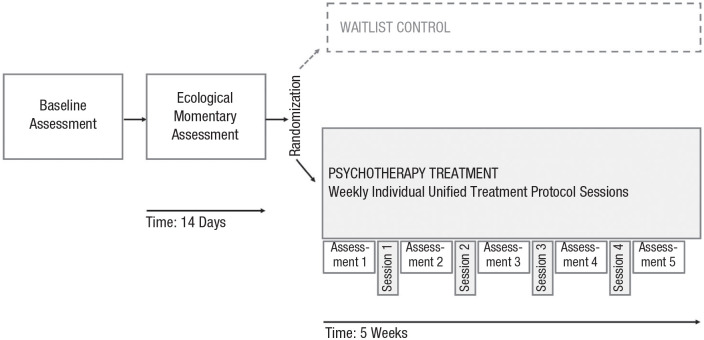
Study design, timeline, and assessments (*N* = 66). For baseline assessments, patients completed a structural clinical interview (Mini International Neuropsychiatric Interview) and self-reported data on demographic variables, general self-efficacy, and clinical, behavioral, and biological samples. For the next 14 days, we prompted patients five times a day on a mobile application installed to their personal phone to assess anxiety experiences, avoidance, hope, and psychophysiological arousal. Patients were then randomly assigned to either psychotherapy treatment (Unified Treatment Protocol) or waitlist-control, depicted as a dashed line (this group was not part of the current analyses). Weekly self-reported anxiety- and depression-symptom-severity assessments were collected before each session. The first assessment during psychotherapy was thus a second baseline assessment because this was obtained before starting psychotherapy. Session-by-session assessments (Assessments 2–5) then followed. Treatment was administered over 16 to 18 weeks in total. Depicted are Sessions 1 through 4 in the psychotherapy-treatment group (*n* = 48) that were included in the current analysis, capturing early treatment change.

The study procedure is depicted in Figure 1.

#### Clinical diagnostic interview

Before participation, eligible individuals received information on procedures and study goals. Participants underwent a telephone screening for the presence of inclusion and exclusion criteria. Individuals eligible for the study gave written informed consent and were invited to a baseline assessment. As part of the OPTIMAX trial (Müller-Bardorff et al., in press), patients completed a structural clinical interview—the Mini International Neuropsychiatric Interview (Lecrubier et al., 1997)—to establish diagnosis. Clinical diagnostic interviews were administered by psychology students on a master level and supervised by trained clinical psychologists. The interrater reliability was .80.

#### Baseline assessments

This assessment comprised several days. Patients provided self-reported data on demographics and personal characteristics, general self-efficacy, clinical (neuro)biological data, and data for memory-related processes in several initial assessment sessions. During the last assessment, patients were instructed about the purpose and proper use of the specially developed mobile application to assess intense repeated measures data in daily life. The app was developed for the Apple iOS platform and for Android smartphones with MobileCoach (www.mobile-coach.eu), an open-source software platform for behavioral interventions and EMAs (Filler et al., 2015; Kowatsch et al., 2017). It was installed on the smartphones of the patients, and a trial assessment was completed. The first assessment day followed on the next day. Patients were prompted five times a day in block-randomized intervals (between 10 a.m. and 8 p.m., divided into five equivalent windows) over a period of 14 days. The application was programmed to prompt just one time and allowed patients to answer the survey within 1 hr after the prompt. The data were coded as missing in case the patients did not respond to the survey within that period. These data were used to assess the effect of self-efficacy and anxiety on avoidance, hope, and perceived psychophysiological arousal. After the 14-day assessment period, the patients were randomly assigned either to the therapy group (UP), for which treatment started immediately, or to the waitlist control group, for which therapy started after 16 weeks. Symptom change was monitored using weekly assessments of symptom severity and impairment in anxiety and depression. Patients rated anxiety and depression symptoms initially during baseline assessment. At the beginning of the first session, anxiety and depression symptoms were assessed a second time, resulting in a second baseline assessment indicating overall symptom severity and impairment in the week before the therapy start. Then, patients received both assessments at the end of each session with the request to rate their symptoms on the evening before the next session. These data were used to assess the effects of self-efficacy on early treatment change.

### Transdiagnostic psychotherapy treatment for anxiety disorders

Patients were treated according to the UP (Barlow et al., 2010; Barlow, Farchione, Sauer-Zavala, et al., 2017) by trained psychotherapists receiving ongoing expert supervision. The treatment includes eight modules: (a) treatment rationale and motivation enhancement, (b) understanding emotions, (c) emotional-awareness training, (d) cognitive appraisal and reappraisal, (e) emotional avoidance, (f) awareness and tolerance of bodily sensations, (g) emotional exposition, and (h) relapse prevention; this treatment was administered over 16 to 18 weeks of individual therapy (60–90 min) on a weekly basis. Therapy sessions included homework and repetition that was accomplished by patients using a personal workbook provided in therapy. The main treatment targets were emotion regulation and its associated mechanisms, such as emotional avoidance, and negative affectivity as the underlying psychological processes of emotional disorders, for example, key to anxiety and affective disorders.

### Measures

#### EMA

Each EMA survey contained 22 items, which were derived from Fisher and Boswell (2016), covering symptoms of anxiety and mood disorders, emotional states, and cognitive and behavioral symptoms, such as avoidance behavior. The patients rated their endorsement of each item in the last 30 min using a visual analog slider ranging from 0 (*not at all*) to 100 (*as much as possible*).

#### Self-efficacy

Perceived self-efficacy was measured using the General Self-Efficacy Scale (Jerusalem & Schwarzer, 1999). On 10 items that use a 4-point Likert scale from 1 (*not at all true*) to 4 (*exactly true*), the patients’ general individual beliefs in their own capacity to overcome hardship are assessed. The total score ranges from 10 to 40; a higher score indicates higher self-efficacy. The internal consistency of the questionnaire was very good (Cronbach’s α = .90).

#### Anxiety symptoms

The Overall Anxiety Severity and Impairment Scale (OASIS; Norman et al., 2006) assesses self-reported anxiety-symptom severity and impairment during the last week on five items using a 4-point Likert scale from 0 (*not at all*) to 4 (*extremely*). This scale has been shown to be sensitive to changes in anxiety during CBT (e.g., Barlow, Farchione, Bullis, et al., 2017) and was assessed weekly before each therapy session in the UP condition. The internal consistency of the OASIS was high at each assessment (Cronbach’s αs = .75–.91).

#### Depression symptoms

Self-reported levels of depression were assessed using the Overall Depression Severity and Impairment Scale (ODSIS; Bentley et al., 2014). The ODSIS captures the severity and impairment of depressive symptoms on five items using a 4-point Likert scale from 0 (*not at all*) to 3 (*extremely*) and is useful for monitoring treatment response (Bentley et al., 2014). The internal consistency of the ODSIS was very high at each assessment (Cronbach’s αs = .90–.97).

### Data analytic strategy

Analyses were conducted using R (Version 3.6.1; R Core Team, 2019) through RStudio IDE (Version 1.2.1335; RStudio Team, 2018) and IBM SPSS (Version 25.0). All analyses were conducted using a Type 1 error rate of α = .05.

#### EMA data preprocessing

We indexed overall anxiety-symptom experiences by six items: avoid activities, leave a situation, avoid people, hopeful, restless, and stressed (Cronbach’s α = .64). We indexed avoidance in daily life using the person mean on each of the three EMA items covering avoidance (avoid activities, leave a situation, and avoid people). To account for the individual avoidance, we used for each patient the avoidance item with the highest score (e.g., the avoidance behavior mostly typical for a particular patient). We indexed hope with a single item derived from Fernandez and colleagues (2017). Such single-item EMA items have recently been shown to have considerable overlap with multiple-item EMA measures and significant predictive validity (Song et al., 2022). Because of a strong and significant correlation between the variables restless and stressed (*r* = .71), we created a composite psychophysiology score by calculating the mean value of the two items, here ranging from 0 to 100; higher scores correspond to greater levels of momentary perceived psychophysiological arousal (Cronbach’s α = .86). Visual inspections of the three outcome variables (avoidance, hope, and psychophysiological arousal) revealed many values at and around 0, which may indicate that respondents did not use the full range of the visual analog slider. We wanted to best account for differences between persons while accounting for this distribution, so we dichotomized the outcome variables at the sample median, which resulted in 50% of the values of the outcome variables having a value of 1 or 0, respectively. In contrast to dichotomizing at the person median, this approach preserves the differences between patients with respect to, for example, avoidance: Patients who indicated stronger avoidance compared with the overall sample are assigned a higher proportion of the value 1 on the new avoidance outcome variable across measurement occasions.

#### Multilevel modeling for multiple observations in baseline assessments

Accounting for the nested structure of the multiple observations within each subject, we used multilevel logistic regression to investigate the relationships between within-subjects observations of EMA data (Level 1) and between-subjects observations of self-efficacy (Level 2). Hence, we described the probability that patients showed avoidance, hope, and psychophysiological arousal as a function of anxiety (Level 1 variable), self-efficacy (Level 2 variable), and their interaction (cross-level interaction) in three multilevel models. The equation of the logistic regression model for patient *j* at time point *i* for each of the three outcome variables is given by:

$$\begin{array}{cc} \frac{p_{ij}}{1-p_{ij}}=\gamma_{00}+\gamma_{10}X_{ij}^{Anxiety}+\gamma_{20}Z_{j}^{SE}+\gamma_{11}X_{ij}^{Anxiety}Z_{j}^{SE} & \left(\mathrm{fixed}\;\mathrm{effects}\right) \end{array}$$

$$\begin{array}{cc} +\;u_{0j}+u_{1j}X_{ij}^{Anxiety} & \left(\mathrm{random}\;\mathrm{effects}\right) \end{array}$$

$$\begin{array}{cc} +\;u_{0j}+u_{1j}X_{ij}^{Anxiety} & \left(\mathrm{random}\;\mathrm{effects}\right) \end{array}$$

$$\begin{array}{cc} +\;\gamma_{30}log(X_{ij}^{Days})\;+u_{3j}log(X_{ij}^{Days}) & \left(\mathrm{control}\;\mathrm{variables}\right) \end{array}$$

Following from this, we investigated whether momentary anxiety $(\gamma_{10}X_{ij}^{Anxiety}),$ trait levels of self-efficacy $(\gamma_{20}Z_{j}^{SE}),$ and their interaction $\left(\gamma_{11}X_{ij}^{Anxiety}Z_{j}^{SE}\right)$ influenced the probability to show momentary avoidance, feelings of hope, and psychophysiological reactions through fixed effects. In addition, we included a random intercept $u_{0j}$ to allow for individual variations in momentary-avoidance, hope, and psychophysiological reactions. The random slopes of the effect of the Level 1 predictor anxiety on these outcome variables $\left(u_{1j}X_{ij}^{Anxiety}\right)$ tested for differences between patients in the effect of anxiety. Days of assessment were entered as a log-transformed predictor variable $(\gamma_{30}log(X_{ij}^{Days}))\;$ that was allowed to vary between patients to examine the individual differences $\left(u_{3j}log(X_{ij}^{Days})\right)$ in symptom development over the assessment period in an exploratory way and to control for residual autocorrelations. Because a linear assumption would be too strong, the log transforms the relation into a negatively accelerated function that is always positive but becomes less steep as the number of days increases. The Level 1 predictor momentary anxiety was person-mean centered and standardized before the analysis to avoid interdependencies because of the inclusion of the main and interaction effects and to facilitate parameter estimation, whereas the Level 2 predictor self-efficacy was grand mean centered. All models were estimated using restricted maximum likelihood estimation and estimated using the *lme4* package (Version 1.1-23; Bates et al., 2015). Statistical significance was assessed with the *lmerTest* package (Version 3.1-2; Kuznetsova et al., 2017). Visualization of the data and plots were created using the *ggplot2* package (Version 3.3.2; Wickham, 2016) and *jtools* package (Version 2.1.0; Long, 2020). Outliers, the normality of residuals, and autocorrelation for the time points were analyzed using the *DHARMa* package (Version 0.3.3.0; Hartig, 2020).

#### Early treatment change

A rationale for the number of sessions defined as “early” in psychological treatments is inconsistent in the literature, ranging from two to eight; previous research has often operationalized early change as the first 4 weeks of treatment (Beard & Delgadillo, 2019). Here, we focus on the first four sessions because the basic modules (treatment rationale, motivation enhancement for treatment engagement, and understanding emotions) of the Unified Treatment Protocol are delivered during these sessions (Barlow, Farchione, Bullis, et al., 2017). We calculated a change score as the difference of the outcome and baseline score to operationalize early symptom change. Because the monitoring of Session 4 was assessed before Session 5, we subtracted scores in anxiety (OASIS) and depression (ODSIS) at the baseline assessment from scores at Session 5. Because the responses of four patients were missing at the second baseline, the baseline scores were used in a linear regression analysis to explore the impact of self-efficacy on this index of early change in symptom severity of anxiety and depression. We computed Cohen’s *f* for the effect size.

## Results

### Descriptive statistics

EMA surveys took an average of 2.94 min per measurement occasion to complete (*SD* = 18.09 min). The completion rate was acceptable (*M* = 63.81%, *SD* = 18.11%) ranging from 23% to 93% of answered EMA surveys per participant. We excluded two participants who provided less than six data points. Within-persons correlations between EMA items ranged from *r* = −.21 (hopeful–psychophysiological arousal) to *r* = .52 (anxious–psychophysiological arousal). Between-persons correlations of EMA items ranged from *r* = −.12 (hopeful—psychophysiological arousal) to *r* = .61 (anxious—psychophysiological arousal; see Table 1). We assessed potential outliers, normality of residuals, and autocorrelation across time points and found no significant violations. The diagnostic groups did not differ in their manifestation of general self-efficacy, *F*(4, 61) = 1.64, *p* = .176 (Fig. 1).

**Table 1.** Demographic and Clinical Sample Characteristics (*N* = 66)

Variable		*n* (%)		*M*		*SD*		Range		*Mdn*
Sex										
	Female	48 (72.7)								
	Male	18 (72.2)								
Age				32.50		11.10		18–58		29
Racial/ethnic identification										
	Swiss	51 (77.3)								
	Non-Swiss European	14 (21.2)								
	Non-European	0 (0)								
	Mixed	1 (1.5)								
Income										
	< 30,000 CHF	9 (13.6)								
	30,000–50,000 CHF	8 (12.1)								
	51,000–70,000 CHF	9 (13.6)								
	71,000–90,000 CHF	22 (33.3)								
	> 90,000 CHF	18 (27.3)								
Education										
	Obligatory school	1 (1.5)								
	High school	20 (30.3)								
	Apprenticeship	19 (28.8)								
	College	4 (6.1)								
	University	22 (33.3)								
States										
	Anxiety			17.91		21.87		0–100		9
	Avoidance			19.88		26.82		0–100		7.5
	Hope			27.11		23.06		0–99		21
	Perceived Psychophysiological arousal			27.96		24.89		0–100		20
Traits and symptoms										
	GSE			25.00		5.59		12–38		24
	BAI			23.91		12.15		1–51		23
	BDI			14.97		5.59		0–38		14
										
Anxiety and depression symptoms during treatment		Baseline					Session 4			
		*n* (%)	*M*	*SD*	Range		*n* (%)	*M*	*SD*	Range
	OASIS		8.84	3.44	2–16			7.42	4.46	0–18
	ODSIS		3.78	4.85	0–15			5.82	4.59	0–17

Note: OASIS and ODSIS symptoms are reported for baseline and Session 4, depicting early change in these symptoms. CHF = Swiss francs; GSE = general self-efficacy; BAI = Beck Anxiety Inventory; BDI = Beck Depression Inventory; OASIS = Overall Anxiety Severity and Impairment Scale; ODSIS = Overall Depression Severity and Impairment Scale.

**Fig. 2.**
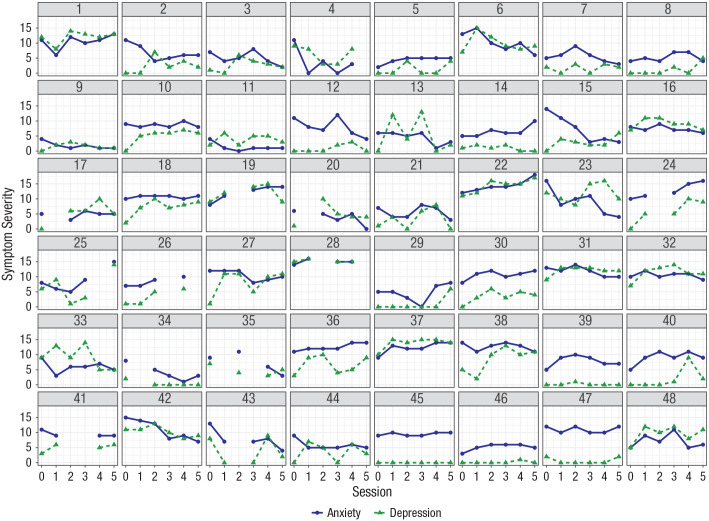
Individual time series of early treatment change in anxiety and depression symptoms. Depicted are changes in anxiety (blue) and depression (green) symptoms across the first five sessions for the 48 patients. Session information: 0 = initial assessment (baseline), 1 = second baseline assessment. The early change score was calculated using the score of Session 5 (indexed after Session 4) to baseline (Session 0). Anxiety severity was assessed with the Overall Anxiety Severity and Impairment Scale (Norman et al., 2006). Depression severity was assessed with the Overall Depression Severity and Impairment Scale (ODSIS; Bentley et al., 2014). Above each panel, patients’ individual anonymized codes are displayed.

Individual trajectories of early treatment change are displayed in Figure 2; 25 patients (55.6%) showed a reduction in anxiety severity after four therapy sessions, and 17 patients (37.8%) showed no early decrease or even an increase in anxiety severity. Average anxiety change was *M* = −1.27 (*SD* = 4.71; range = −12 to 7). For early change in depression, eight patients (17.8%) showed a reduction after four therapy sessions, and 29 patients (64.4%) showed an increased score in depression severity. The average change in depression severity was *M* = 2.36 (*SD* = 3.49; range = −6 to 10; see Table 1).

### Moderating effects of self-efficacy on anxiety effects in patients’ daily lives

As outlined above, a random intercept random slope model with interactions between self-efficacy and anxiety was fitted and interpreted for each of the three outcome variables (Table 2).

**Table 2.** Main and Interaction Effects of Anxiety, Self-Efficacy, and Time (Days) on Avoidance, Hope, and Perceived Psychophysiological Arousal

	Avoidance				Hope				Perceived psychophysiological arousal			
Fixed effects	*b*	*OR*	[95% CI]	*z*	*b*	*OR*	[95% CI]	*z*	*b*	*OR*	[95% CI]	*z*
(Intercept)	0.09	1.09	[0.68, 1.76]	0.36	0.31	1.37	[0.83, 2.24]	1.24	0.84	2.31**	[1.24, 4.29]	2.65
Anxiety	0.43	1.54***	[1.34, 1.76]	6.26	–0.41	0.66***	[0.57, 0.77]	–5.23	1.18	3.24***	[2.54, 4.14]	9.47
GSE	–0.63	0.53**	[0.34, 0.84]	–2.68	0.28	1.32	[0.82, 2.13]	1.14	–0.68	0.51*	[0.28, 0.92]	–2.24
Anxiety × GSE interaction	–0.11	0.90	[0.78, 1.03]	–1.50	0.19*	1.21*	[1.03, 1.42]	2.37	–0.24	0.79*	[0.62, 1.00]	–2.00
Days	0.06	1.06	[0.87, 1.28]	0.58	–0.23	0.80	[0.62, 1.02]	–1.83	–0.39	0.67***	[0.54, 0.84]	–3.48
Random effects	*SD*				*SD*				*SD*			
$u_{0j}$	1.68				1.74				2.25			
$\varepsilon_{ij}$	1.81				1.81				1.81			
$u_{1j}$	0.34				0.43				0.74			
$u_{3j}$	0.56				0.81				0.66			

Note. *OR* = odds ratio; CI *=* confidence interval; GSE = general self-efficacy; Anxiety × GSE interaction = the interaction between the momentary anxiety and GSE; days = exploratory predictor variable to quantify assessment period (14 days in total); *b* = unstandardized regression coefficient; *z* = *z* score of binomial distribution; $\varepsilon_{ij}$ = within-persons residual variance; $u_{0j}$ = between-persons variance; $u_{1j}$ = within-persons variance of anxiety; $u_{3j}$ = within-persons variance.

* *p* < .05. **p < .01. ***p < .001.

#### Avoidance

**Fig. 3.**
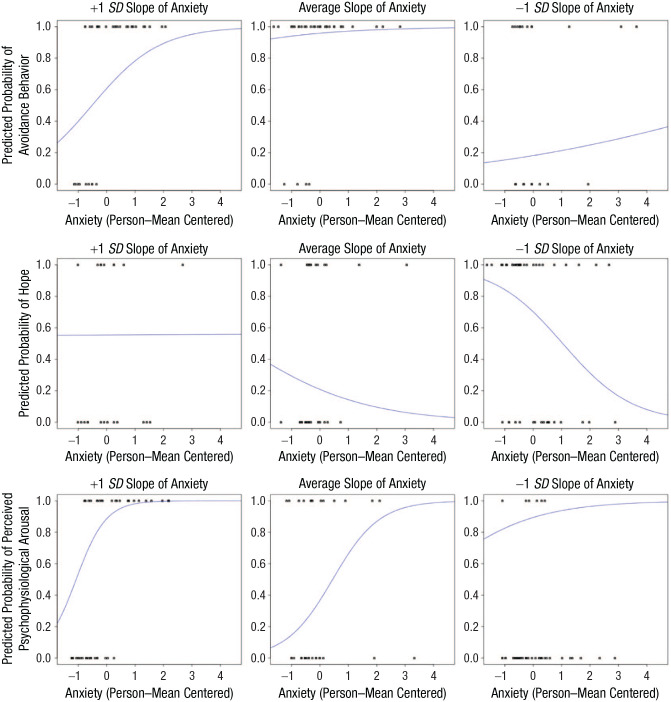
Variability of individual slopes for anxiety and avoidance, hope, and perceived psychophysiological arousal. Individual slopes of anxiety for three exemplary patients on the predicted probability of (top) avoidance, (middle) hope, and (bottom) perceived psychophysiological arousal in daily life are displayed. Anxiety is person-mean centered. (Left) Patient with a +1 *SD* slope of anxiety. (Middle) Patient with an average slope of anxiety. (Right) Patient with a −1 *SD* slope of anxiety. Slopes have a sigmoid shape because of the logistic regression model.

**Fig. 4.**
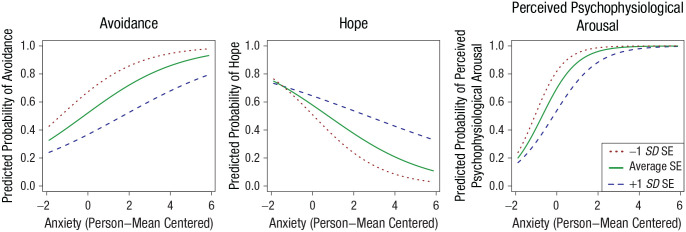
Interactions of anxiety and self-efficacy on avoidance, hope, and perceived psychophysiological arousal. Anxious = person-mean centered and standardized; GSE = self-efficacy (grand mean centered); avoidance, hope, perceived psychophysiological arousal = dichotomizedSlopes have a sigmoid shape because of the logistic regression model. (Left) Joint effects of anxiety and GSE on the probability of showing avoidance. The main effect of anxiety is visible because the curves have positive slopes, whereas the main effect of GSE is visible through the order of the curves (e.g., the curve for +1 *SD* lies below all other curves). The parallel curves show that there is no interaction effect. (Middle) Main effect of anxiety through negative slopes and the interaction effect shows that slopes are not equal across levels of GSE. (Right) Positive main effect of anxiety, negative main effect of GSE, and their interactions are visible because the curves are not parallel.

Anxiety showed a significant effect on avoidance across patients (odds ratio [*OR*] = 1.54, 95% CI = [1.34, 1.76], *p* < .001), indicating that avoidance became more likely as concurrent anxiety increased. There was substantial variation of the effect of anxiety on avoidance between patients; 68% of effects were in the interval *b* = 0.43 ± 0.34 (Fig. 3, top). The main effect of self-efficacy on avoidance was significant, indicating that individuals who exhibited lower levels of self-efficacy at baseline were more likely to avoid during the EMA period (*OR* = 0.53, 95% CI = [0.34, 0.84], *p* = .008; Fig. 4, left). However, we found no effect of time on avoidance.

#### Hope

Anxiety showed a significant effect on hope across patients (*OR* = 0.66, 95% CI = [0.57, 0.77], *p* < .001), indicating that as anxiety increased, individuals exhibited concurrently decreased levels of hope. There was substantial variation of the effect of anxiety on feeling hopeful between patients; 68% of the effects were in the interval *b* = −0.41 ± 0.43 (see Fig. 3, middle). There was no main effect of self-efficacy on hope and no effect of time. The cross-level interaction between anxiety and self-efficacy was significant such that individuals who exhibited lower levels of self-efficacy at baseline were more likely to lose hope as concurrent anxiety increased during the EMA period (*OR* = 1.21, 95% CI = [1.03, 1.42], *p* = .02). All effects are displayed in Figure 4 (middle).

#### Perceived psychophysiological arousal

Anxiety showed a significant effect on perceived psychophysiological arousal across patients (*OR* = 3.24, 95% CI = [2.54, 4.14], *p* < .001), indicating that perceived psychophysiological arousal became more likely as concurrent anxiety increased. There was substantial variation of the effect of anxiety on perceived psychophysiological arousal between patients; 68% of the effects were in the interval *b* = 1.18 ± 0.74 (see Fig. 3, bottom). The main effect of self-efficacy on perceived psychophysiological arousal was significant, indicating that perceived psychophysiological arousal became more likely the lower someone’s self-efficacy was at baseline assessment (*OR* = 0.51, 95% CI = [0.28, 0.92], *p* = .025). There was a significant cross-level interaction between anxiety and self-efficacy such that individuals who exhibited lower levels of self-efficacy at baseline were more likely to exhibit perceived psychophysiological arousal as concurrent anxiety increased during the EMA period (*OR* = 0.79, 95% CI = [0.62, 1.00], *p* = .046; see Fig. 4, right). Assessment day significantly predicted the probability of perceived psychophysiological arousal (*OR* = 0.67, 95% CI = [0.54, 0.84], *p* < .001; see Fig. 2).

#### Self-efficacy and early symptom change

Self-efficacy at baseline had a significant effect on early anxiety change, *F*(1, 43) = 2.96, *p* = .045, *f* = 0.25; *b* = −0.25, *t*(43) = −0.72, *p* = .045 (see Fig. 3). Patients reporting higher self-efficacy showed a greater decrease in anxiety symptoms early in treatment. The effect on depression change was nonsignificant (*p* > .05).

## Discussion

In this study, we investigated effects of self-efficacy on avoidance, hope, and perceived psychophysiological arousal in daily life in patients with anxiety disorders. To the best of our knowledge, our study is the first to show that in patients’ daily lives, individual differences in self-efficacy significantly predict the likelihood of daily avoidance behavior. Self-efficacy also buffered the association between anxiety, hope, and perceived psychophysiological reactions. Individuals high in self-efficacy showed more hope and perceived fewer psychophysiological reactions in moments of higher anxiety. We then examined the effects of self-efficacy on early symptom change during transdiagnostic CBT treatment. Significant effects of self-efficacy on early treatment change during a transdiagnostic treatment emerged for anxiety but not for depression.

In our sample of patients with anxiety disorders, we found significant interindividual variability in general self-efficacy. Higher levels of self-efficacy decreased the likelihood of avoiding persons, situations, and places in daily life, whereas higher momentary anxiety increased such behavior. More self-efficacious patients might be more likely to initiate and face feared situations in daily life and, in turn, benefit from new learning about their own abilities and the feared situations because of these experiences. This is in line with recent experimental research that demonstrated how low perceived self-efficacy altered cognitive and expectancy components of discrimination during fear learning and hampered individuals’ ability to learn associations between ambiguous cues and threat and safety learning (Raeder et al., 2019). Such reduced ability to discriminate between safety and danger cues might also reduce the efficacy of exposure-based treatment. Recent research indeed linked the effects of self-efficacy with better outcomes after exposure therapy (Böhnlein et al., 2020). Neurobiologically, these mechanisms and effects of self-efficacy could be related to the glutamatergic system, which has been proposed to be a key player in anxiety and associated with higher perceived lack of control and elevated glutamatergic response associated with stressor-avoidance behavior (Bryant et al., 2014; Cortese & Phan, 2005).

Self-efficacy did not interact with anxiety to predict avoidance. Different reasons may account for this finding. We indexed general self-efficacy before EMA assessments, which might be more predictive of trait avoidance than avoidance in specific situations, including safety behaviors in unique situations (Kirk et al., 2019). Patients might also not have responded to EMA prompts in particularly stressful situations in their daily lives because answering these questions might be difficult to perform while avoiding at the same time, and avoidance might thus not have been fully captured during such critical times. Objective measures or avoidance proxies, for example, via GPS monitoring and passive mobile sensing, might provide additional data and help circumvent potential self-reporting biases and missing data; such tools would be useful to capture avoidance behavior in daily life (Chow et al., 2017; Onnela, 2020).

Momentary anxiety was related to feeling less hopeful at the same time. As hypothesized, general self-efficacy explained some differences in this relationship between anxiety and hope. In contrast to patients with lower self-efficacy who are less likely to experience hope when feeling anxious, patients with higher levels of self-efficacy were more likely to experience hope when feeling anxious. This is in line with the impact of positive expectations on changes in symptoms and psychopathology (Constantino et al., 2011; Meyerhoff & Rohan, 2016). Self-efficacy might have initiated hope and supported patients in dealing with stressful situations in daily life. Previous studies have suggested that the relation between positive and negative affect—here, feeling hopeful and feeling anxious—shifts from relative independence toward more dependent relationships during personally relevant stressful events (Dejonckheere et al., 2019; Zautra et al., 2005). Self-efficacy could be a protective factor supporting patients in coping with personally relevant situational demands. Enhancing self-efficacy during psychotherapy, especially when confronting patients with negative emotions or situations, could be a promising strategy to help patients face difficult situations, increase hope, and lead to better outcomes (Gallagher et al., 2019, 2020). This is in line with Albert Bandura’s social-cognitive theory, according to which individuals are agents who engage proactively in their own development, adaption, and change, that is, are capable to influence their life circumstances. This belief in one’s own efficacy (self-efficacy) constitutes a core mechanism of human agency and posits the belief to achieve goals and exercise control over one’s environments, thoughts, emotions, and actions. Thus, self-efficacy describes a generative ability that coordinates and incorporates cognitive, social, emotional, and behavioral skills to undertake appropriate activities under varying situational demands. Accordingly, self-efficacy builds a basic premise for human behavior, motivation, performance accomplishments, and emotional well-being (Bandura, 1977; Maddux & DuCharme, 1997).

Momentary anxiety was positively related—although self-efficacy was negatively related—to the likelihood of self-reported psychophysiological arousal in daily life. Patients with lower self-efficacy were more likely to experience psychophysiological arousal when feeling anxious. Such beneficial effects of self-efficacy have previously been shown in the laboratory such that experimentally induced self-efficacy had effects on interoception of arousal (Paersch et al., 2021) and autonomic arousal, including decreased heart rate and skin-conductance levels (Bandura et al., 1988; Zlomuzica et al., 2015). Self-efficacy was mostly effective here when anxiety levels were below an individual’s average, but not under high-anxiety levels, which indicates limits of socio-cognitive protective factors and has clinical implication. Cognitive strategies might be more feasible under lower anxiety levels, although in higher levels of anxiety in daily life, other strategies focused on reducing bodily reactions might first be more indicated. We also found that the later an assessment occurred during the 14-day assessment period, the less likely a patient was to experience perceived psychophysiological arousal in daily life, with slopes varying individually. There was no such effect of assessment days for avoidance or hope. Reactivity during longitudinal monitoring of one’s emotional states could be one explanation for this (Conner & Reid, 2012; De Vuyst et al., 2019), albeit explanations would be warranted regarding specificity of such effects on arousal assessed in the diary.

As expected, self-efficacy affected early treatment change in anxiety symptoms. This is in line with previous research that proposed baseline self-efficacy as a predictor of treatment success (Jakubovski & Bloch, 2016). Our results expand these findings to the initial period of psychotherapy and trajectories associated with better outcomes over time (Beard & Delgadillo, 2019; Stulz et al., 2007). Although some studies have suggested rapid cognitive changes during the first half of CBT treatment in some patients, including self-efficacy (Casey et al., 2005; Keshen et al., 2017), others have reported the greatest changes in self-efficacy during later stages of treatment (Fentz et al., 2013; Gallagher et al., 2013). Here, we corroborate the effects for early change in psychotherapy. Self-efficacy has been suggested to influence cognitive processes, such as goal setting and commitment, and motivational processes (Bandura, 1994), and these were part of the first sessions in the treatment. More self-efficacious patients might have engaged more actively during the first treatment sessions, set more specific goals, and translated the treatment content to their daily lives and symptoms more effectively, as documented by the EMA. Future research should examine within-persons changes of self-efficacy over the course of therapy on a weekly basis to describe these individual processes and bidirectional associations between levels of perceived self-efficacy, treatment engagement and motivation, and symptom severity (Casey et al., 2005; Vincent & Norton, 2019).

Our study is not without limitations. First, because of nonnormal distribution of the outcome variables, we dichotomized variables at the sample median, which resulted in 50% of the values of the outcome variables having a value of 1 or 0, respectively. Consequently, our measurements are conflated by between-subjects variance in likelihood. Second, a larger sample size would have allowed for an investigation into the individual trajectories of symptom change during the first sessions of treatment and their associations with self-efficacy. Third, this study did not have a healthy control group as a comparator. This potentially limits conclusions about the specificity of the results for anxiety disorder versus mental disorders in general. Thus, this should be considered when interpreting the results. Fourth, EMA was conducted between the hours of 10 a.m. and 8 p.m. and may not have fully captured all relevant processes in the early morning, given that anxiety levels might be higher in the mornings (Helbig-Lang et al., 2012; Kenardy et al., 1992). Fifth, we examined patients with mixed anxiety symptoms and employed a transdiagnostic treatment program. Our findings should be replicated in other samples, including samples comprising specific anxiety disorders and in the context of other CBT programs. Sixth, participants were recruited from the community and not clinic referred, which might limit generalizability of our results. However, recruitment ads were explicitly directed at patients who wanted to undergo therapy for anxiety disorders, and participants all met diagnostic criteria for an anxiety disorder. Finally, we did not obtain information on culture and geographic background, which should be included in future studies.

Despite these limitations, our study provides novel insights into the association between self-efficacy and within-persons dynamics of symptoms instead of relying on between-persons differences in anxiety disorders. There was significant and meaningful variability in self-efficacy of patients with an anxiety disorder, and self-efficacy influenced patterns of behavior and experiences in daily life. These idiographic approaches might contribute to personalized diagnostic approaches and case conceptualization to identify and target specific mechanisms at the individual level (Fisher, 2015; Fisher et al., 2019).

Future research should evaluate the potential of enhancing self-efficacy during treatment with specific interventions. Although enhancing self-efficacy is often an implicit target across the entire course of CBT, interventions could be added to elicit self-efficacy continuously throughout treatment, for instance, by applying homework assignments to practice and master specific skills and generalize to daily life. Furthermore, some studies have suggested brief and experimental ways to enhance self-efficacy, for instance, by recalling autobiographical self-efficacy memory episodes (Paersch et al., 2021). Such interventions could be integrated into existing treatment plans and used to personalize and augment the treatments for patients with anxiety disorders. Digital technology, such as wearables, provides a promising addition to deliver treatments and additionally collect objective markers of psychopathology, for instance, to assess patients’ avoidance, a behavior that is challenging to capture even with self-reported diary assessments (e.g., Onnela, 2020).

## Transparency

*Action Editor:* Jennifer Lau

*Editor:* Jennifer L. Tackett

*Author Contributions*

**Christina Paersch:** Conceptualization; Data curation; Formal analysis; Investigation; Methodology; Project administration; Writing – original draft; Writing – review & editing.

**Dominique Recher:** Conceptualization; Data curation; Formal analysis; Investigation; Methodology; Project administration.

**Ava Schulz:** Conceptualization; Data curation; Project administration.

**Mirka Heininger:** Data curation; Formal analysis; Investigation; Methodology; Supervision; Validation; Visualization; Writing – original draft; Writing – review & editing.

**Barbara Schlup:** Supervision.

**Florian Kuenzler:** Methodology; Software.

**Stephanie Homan:** Data curation; Formal analysis; Investigation; Methodology; Supervision; Visualization; Writing – original draft; Writing – review & editing.

**Tobias Kowatsch:** Conceptualization; Funding acquisition; Supervision; Validation; Writing – original draft; Writing – review & editing.

**Aaron J. Fisher:** Conceptualization; Formal analysis; Investigation; Supervision; Validation; Writing – original draft; Writing – review & editing.

**Andrea B. Horn:** Formal analysis; Investigation; Writing – original draft; Writing – review & editing.

**Birgit Kleim:** Conceptualization; Funding acquisition; Investigation; Project administration; Resources; Supervision; Validation; Writing – original draft; Writing – review & editing.
